# Anti-Cataract Effect of the Traditional Aqueous Extract of Yerba Mate (*Ilex paraguariensis* A. St.-Hil.): An In Ovo Perspective

**DOI:** 10.3390/life14080994

**Published:** 2024-08-10

**Authors:** Seyma Oncu, Merve Becit-Kizilkaya, Abdulkadir Bilir, Alperen Saritas, Evrim Suna Arikan-Soylemez, Halit Bugra Koca, Fatma Firat, Afife Busra Ugur-Kaplan, Mustafa Abdullah Yilmaz

**Affiliations:** 1 Department of Medical Pharmacology, Faculty of Medicine, Afyonkarahisar Health Sciences University, Afyonkarahisar 03030, Turkey; 2 Department of Pharmaceutical Toxicology, Faculty of Pharmacy, Afyonkarahisar Health Sciences University, Afyonkarahisar 03030, Turkey; mervebecit@hotmail.com; 3 Department of Anatomy, Faculty of Medicine, Afyonkarahisar Health Sciences University, Afyonkarahisar 03030, Turkeyalperen.saritas@hotmail.com (A.S.); 4 Department of Medical Biology, Faculty of Medicine, Afyonkarahisar Health Sciences University, Afyonkarahisar 03030, Turkey; arikanmt@gmail.com; 5 Department of Biochemistry, Faculty of Medicine, Afyonkarahisar Health Sciences University, Afyonkarahisar 03030, Turkey; bugrakoca@yahoo.com; 6 Department of Histology and Embryology, Medicine Faculty, Afyonkarahisar Health Sciences University, Afyonkarahisar 03030, Turkey; fatmaozturk87@gmail.com; 7 Department of Pharmaceutical Technology, Faculty of Pharmacy, Ataturk University, Erzurum 25240, Turkey; afife.busra.ugur@gmail.com; 8 Department of Analytical Chemistry, Faculty of Pharmacy, Dicle University, Diyarbakir 21280, Turkey; mustafaabdullahyilmaz@gmail.com

**Keywords:** *Ilex paraguariensis*, yerba mate, cataract, chick embryo, *CRYAA*

## Abstract

Introduction: The therapeutic effect of different doses of the traditional aqueous extract of dried leaves of yerba mate (*Ilex paraguariensis* A. St.-Hil.) was investigated in an experimental cataract model in chicken embryos. Methods and Results: LC-MS/MS analysis allowed the identification and quantification of 53 metabolites. In the hydrocortisone-induced cataract model, lenses were examined morphologically after treatment and parameters related to oxidative stress (total antioxidant/oxidant status (TAS/TOS), glutathione (GSH), and malondialdehyde (MDA)) were evaluated. Antiproliferative cell nuclear antigen (PCNA) and caspase-3 H-scores were determined and crystallin alpha A (*CRYAA*) gene expression in the lenses was measured by RT-PCR. The degree of cataract decreased in all treatment groups. While there was no significant difference in TAS levels compared to the negative control, TOS, GSH, and MDA levels were dose-dependently regulated. Treatment groups other than the high-dose group regulated the decrease in PCNA and the increase in caspase-3. *CRYAA* gene expression increased significantly only at the lowest dose. Conclusion: YM, which is becoming increasingly popular as a traditional tea, showed a therapeutic effect on hydrocortisone-induced cataracts in chicken embryos at relatively low doses.

## 1. Introduction

A cataract occurs when the lens of the eye loses its permeability to light and visual acuity decreases. Today, cataracts are the most common cause of reversible vision loss around the world. The etiopathogenesis of the disease is not yet fully understood, but various factors such as aging, diabetes, long-term steroid treatment, dietary habits, smoking, alcohol consumption, and sun exposure play a role in the development of cataracts [1]. In particular, oxidative damage to proteins in the lens is an important hypothesis [2,3].

Conservative and surgical methods are used in the treatment of cataracts. Although ophthalmic surgery is an effective and safe method, it has its limitations as it is expensive and involves complications and surgical treatment is not easily accessible in every country. Therefore, cheap, safe, and effective alternative methods are needed to prevent cataracts or delay their development [4,5]. Given the active role of oxidative stress in the development of cataracts, the consumption of natural antioxidant substances with high activity and low side effects is therefore thought to be a good alternative for this purpose [6,7].

The dried leaves *Ilex paraguariensis* A. St.-Hil. (Yerba mate, YM), which are known for their strong antioxidant effect, are often consumed in the form of infusions (tea) in South American countries. YM, also known as Paraguayan tea, is becoming increasingly popular in North America and Europe [8,9]. The leaves of YM are rich in alkaloids, flavonoids, and phenols. These compounds are responsible for the bioactivity of YM, which includes antioxidant, anti-inflammatory, antimutagenic, and hypoglycemic properties [10,11,12,13]. Due to the increasing popularity of YM, a recent study determined the antioxidant activities of the extract obtained from the leaves using four well-known in vitro techniques (DPPH, FRAP, TPC, and TFC). The result of this study was that the extract showed high antioxidant activity even at very low concentrations [12].

Despite some limitations, such as the differences between species, the different absorption rate of chemicals, and high costs, laboratory animals such as mice and rats are often used in experimental research. The embryonic chick model is considered a better alternative to mice and rats due to its easy accessibility, reproducibility, and low cost. The chick model has recently become increasingly important [14]. The hydrocortisone (HC)-induced cataract model in chicken embryos is a suitable and ideal model for simulating the disease [15]. Therefore, the steroid-induced cataract model in chicken embryos used in this study is very practical because it is easy to develop and allows the treatment and screening of drugs with antioxidant activity [16]. Previously, many antioxidant compounds such as astaxanthin [17], vitamin E [18], piperine [2], betaine [19], and ginsenoid-Rb1 [20] have been reported to suppress cataract formation in this model.

We found no experimental or clinical studies investigating the protective effect of YM aqueous extract against cataracts. In this study, we performed the identification and quantification of phenolic compounds in the extract by liquid chromatography–tandem mass spectrometry (LC-MS/MS) and evaluated the potential protective effect of YM aqueous extract against HC-induced cataracts in chicken embryos by morphological, biochemical, and immunohistochemical methods.

## 2. Experimental Section

### 2.1. Chemicals and Plant

The dried leaves of *Ilex paraguariensis* A. St.-Hil. were purchased from Fitovision Medical Herbal Tea (Antalya, Turkey, serial no: SC114) in October 2022. Standard compounds for LC-MS/MS were purchased from Merck (Darmstadt, Germany) and Sigma Aldrich (St. Louis, MO, USA). HC (100 mg/5 mL, Hydrocort-liyo) was purchased from Kocak Pharma (Kocaeli, Turkey).

### 2.2. Plant Extraction

The extract was prepared according to the traditional infusion method. The powdered drug was mixed with boiled water (1:10 *w*/*v*) and stirred in a magnetic stirrer until the temperature dropped to 40 °C. After filtration, the excess water was evaporated at 45 °C using a Heidolph 4001 Efficient evaporator (Schwabach, Germany). The resulting extract was then subjected to freeze-drying, which involved freezing the extract overnight at −20 °C and lyophilizing it at 0.021 mbar at −55 °C using a Martin Christ Alpha 1-2 LD Plus freeze dryer (Osterode, Germany). The freeze-dried extract was stored at −20 °C and protected from light for further studies [21].

### 2.3. Determination of Phenolic Compounds

The phytochemical analysis of the YM aqueous extract was performed using an LC-MS/MS method developed and validated in a previous study by Yilmaz [22]. The analysis was carried out using a Shidmadzu-Nexera model ultra-high performance liquid chromatography (UHPLC) system coupled with a tandem mass spectrometer. The reversed-phase UHPLC was equipped with an autosampler (model SIL-30 AC), a column oven (model CTO-10ASvp), binary pumps (model LC-30 CE), and a degasser (model DGU-20A3R).

### 2.4. Experimental Animals and Study Design

The animal experiments were performed after approval by the Local Ethics Committee for Animal Experiments of Afyon Kocatepe University (approval no. 49533702/48, date: 6 April 2022). All experiments were performed in accordance with the animal experiment protocol.

In this study, 0-day-old fertilized White Leghorn specific pathogen-free (SPF) chicken eggs (60 ± 5 g, n = 100) were obtained from commercial broiler rootstocks (Konya, Turkey). The eggs were incubated for 17 days at 70% relative humidity and 37.5 °C. Based on a previously described method [15], saline/HC/YM extract was injected into the air sac of the eggs using insulin injectors on the 15th day of incubation [23]. The groups (n = 20) were divided as follows:

Control group (C): Eggs were injected with 100 µL (isotonic saline (0.9%), which served as a negative control.

Hydrocortisone group (HC): Eggs were injected with HC (0.50 µmol/100 µL), which served as a positive control.

YM treatment groups: Eggs were treated with YM at different doses (YM-1: 62.5 mg/kg, YM-2: 125 mg/kg, and YM-3: 250 mg/kg), 3 h after the injection of HC (0.50 µmol/100 µL).

After injection, the puncture was sealed with sterile cellophane tape and on the 17th day of incubation, all chick embryos were removed from the eggs, and their eyes were dissected. The dissected eyes were divided into three groups for further analysis: (I) Seven intact pairs of eyes were preserved in formalin for histopathological examination. (II) Eight pairs of eyes were examined for biochemical analysis after an assessment of lens opacity. The assessment was evaluated with reference to previous studies [2,23]. (III) The remaining lenses were stored in eppendorf tubes at −20 °C for use in genetic analysis later.

### 2.5. Biochemical Analysis

The storage conditions and homogenization of the lens samples were similar to the previous study [24]. The following biochemical parameters were measured in the lens homogenates. The total antioxidant status (TAS) and total oxidant status (TOS) were measured colorimetrically using a commercial kit (Rel Assay Diagnostic, Mega Medical, Gaziantep, Turkey). TAS and TOS values were expressed as µmol Trolox-equiv/lens and µmol H_2_O_2_-equiv/lens, respectively. Malondialdehyde (MDA) and glutathione (GSH) levels were determined using commercially available ELISA kits (BT LAB, Shanghai, China). GSH and MDA levels were expressed as nmol/lens and mg/lens, respectively. All absorbance experiments were performed using a microanalyzer (ChemWell 2910, Awareness Technology Inc., Martin Hwy., Palm City, FL, USA).

### 2.6. Immunohistochemical Analysis

After routine histologic preparation of the eye specimens (embedded in paraffin), 5 micrometer sections of the largest surface of the lens were prepared on adhesive slides. All lenses were positioned in the same direction during embedding and sections were made from all lenses in the same plane. H-score analyses were performed on the sections closest to the midsagittal sections, where the cell density is highest. Then, 500 cells from 7 different areas of each tissue were counted for each lens and the arithmetic means were taken. The H-score was calculated using the formula “(5 × the number of very strongly staining cells) + (4 × the number of strongly staining cells) + (3 × the number of moderately staining cells) + (2 × the number of weakly staining cells) + (1 × the number of very weakly staining cells) ≤ 500”.

The prepared samples were stained with antiproliferative cell nuclear antigen (PCNA) (Novus, NB500-106, Littleton, CO, USA) to assess proliferation and caspase-3 (Novus-NB100-56708) to assess apoptosis (antibodies are compatible with chick). Chromogenic DAB substrate was used for immunohistochemical staining. Nuclear counterstaining was performed with Mayer’s hematoxylin. For immunohistochemical staining, the application steps in Turan and Turan’s [25] studies for PCNA staining and Kundakci et al.’s study [20] for caspase-3 staining were performed according to the manufacturer’s instructions.

### 2.7. Genetic Analysis

The mRNA content of crystallin alphaA (*CRYAA*) in the treatment samples was analyzed by the real-time PCR using the Step-One-Plus thermal cycler (Applied Biosystems, Forster City, CA, USA). The total reaction volume for amplifications was 20 μL, which included the following components: cDNA template, site-specific primers (Oligomer Biotechnology, Ankara, Turkey) (Table 1), SsoAdvanced Universal Inhibitor-Tolerant SYBR Green Supermix (Biorad, Hercules, CA, USA, Cat. No.: 172-50-16), and nuclease-free water. The dissociation curve of the real-time PCR is also shown in the Supplementary Figure S1. The amplification reactions were carried out using these components in the thermal cycler, following the manufacturer’s instructions.

### 2.8. Statistical Analysis

Statistical analysis was performed using SPSS v.24.0 (SPSS Inc., Chicago, IL, USA). Comparisons between different groups were analyzed using the Kruskal–Wallis test, followed by post-hoc tests, and the Dunn test with a Bonferroni correction. The Pearson chi-square test was used to analyze differences between categorical variables such as cataract stages and groups. A *p*-value of less than 0.05 was considered statistically significant for all analyses.

## 3. Results

### 3.1. Phytochemical Compounds of YM Extract Using LC-MS/MS

**Figure 1:**
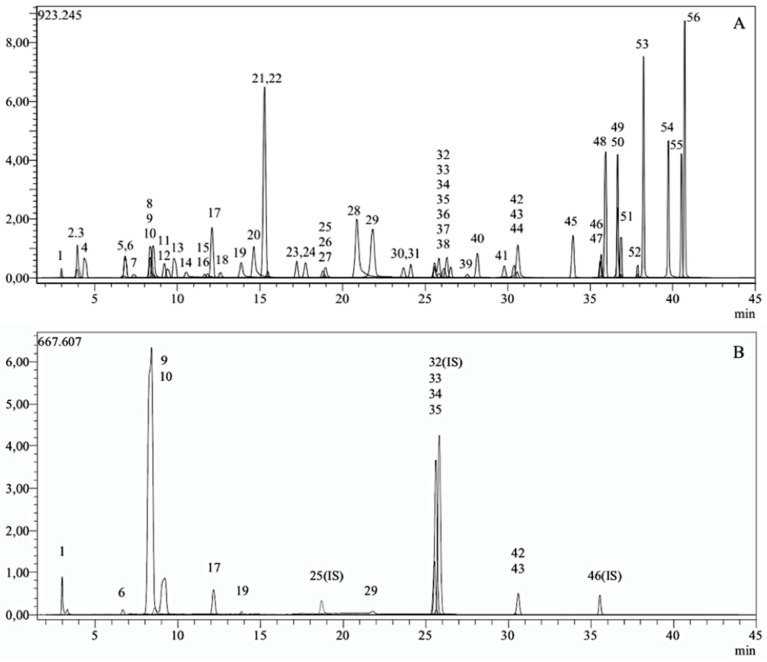
LC-MS/MS chromatograms of standards (**A**) and YM (**B**). YM: yerba mate aqueous extract.

Figure 1 illustrates the standard chromatogram of the phytochemical compounds. The method employed 53 phytochemical compounds as standards, and the presence of the 12 compounds in the extract was confirmed by comparative chromatography with reference standards (Table 2).

### 3.2. Grading of Cataracts

**Figure 2:**
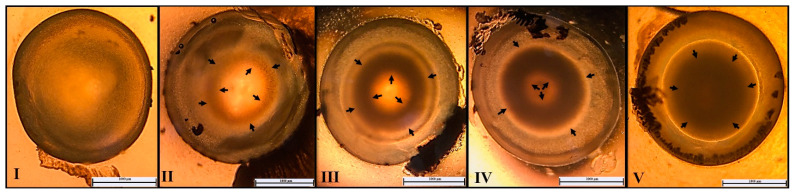
Images of cataract degrees of the groups. **I**: grade 1, **II:** grade 2, **III:** grade 3, **IV:** grade 4, **V:** grade 5.

A total of 80 lenses, 16 lenses in each group, were examined under a stereomicroscope to determine the grade of cataract (Figure 2). No cataract was observed in any lens in group C, while all lenses in group HC showed cataract formation and the mean cataract grade was 3.94. The YM treatment groups (low, medium, and high doses) showed varying degrees of cataract formation at 3.6, 2.6, and 3, respectively (Table 3). A significant difference in cataract grade was observed between groups C and HC (*p* < 0.05). Compared to HC, a significant decrease in cataract grades was observed in treatment groups YM-2 and YM-3 (*p* < 0.05).

### 3.3. Biochemical Analysis

**Figure 3:**
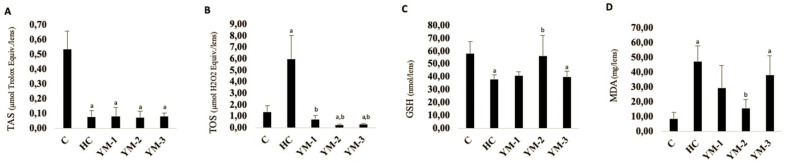
Effect of YM on oxidative stress markers in lenses. TAS (**A**), TOS (**B**), GSH (**C**) and MDA (**D**). Data are expressed as the means ± SD, **^a^** *p* < 0.05 vs. C, **^b^** *p* < 0.05 vs. HC. **C:** control group, **HC:** hydrocortisone group, **YM:** yerba mate aqueous extract, **YM-1:** YM 62.5 mg/kg group, **YM-2:** YM 125 mg/kg group, **YM-3:** YM 250 mg/kg group.

Figure 3 shows the mean values of TAS, TOS, GSH, and MDA in the lenses of the experimental groups. All investigated parameters showed significant statistical differences between groups C and HC (*p* < 0.05). The YM-treated groups exhibited varying differences compared to HC, depending on the dose administered and the specific parameters assessed.

### 3.4. Immunohistochemical Analysis

**Figure 4:**
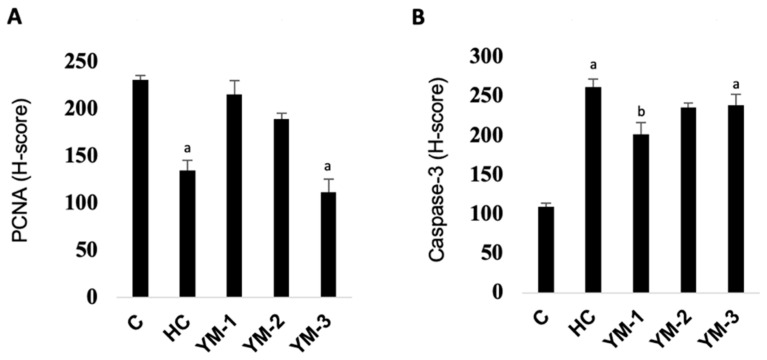
Effect of YM on PCNA (**A**) and Caspase-3 H (**B**) in lenses. Data are expressed as the means ± SD, **^a^** *p* < 0.05 vs. C, **^b^** *p* < 0.05 vs. HC. **C:** control group, **HC:** hydrocortisone group, **YM:** yerba mate aqueous extract, **YM-1:** YM 62.5 mg/kg group, **YM-2:** YM 125 mg/kg group, **YM-3:** YM 250 mg/kg group.

**Figure 5:**
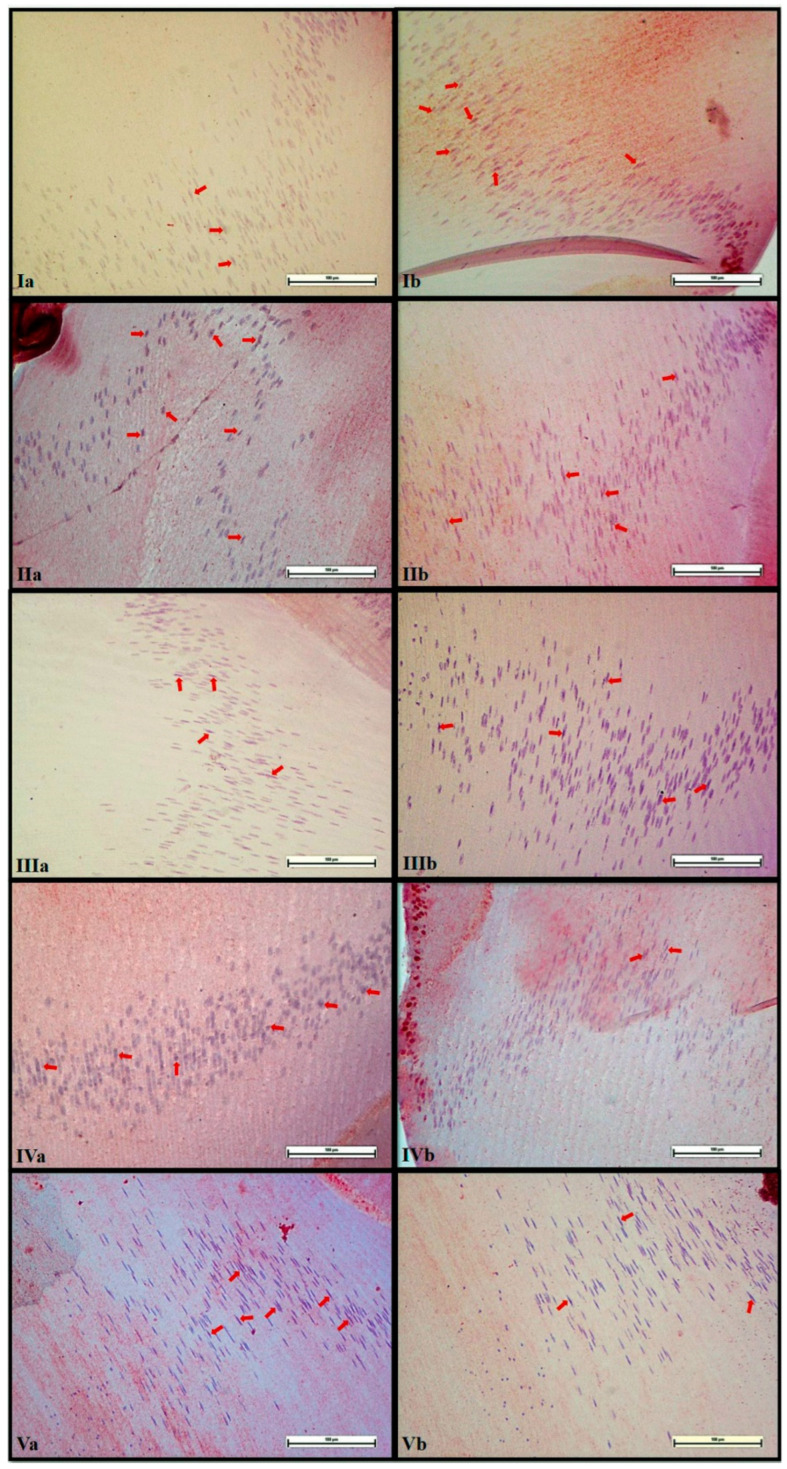
Caspase-3 (**a**) and PCNA (**b**) staining in lenses. **I:** C, **II:** HC, **III:** YM-1, **IV:** YM-2, **V:** YM-3. **C:** control group, **HC:** hydrocortisone group, **YM:** yerba mate aqueous extract, **YM-1:** YM 62.5 mg/kg group, **YM-2:** YM 125 mg/kg group, **YM-3:** YM 250 mg/kg group.

The mean PCNA and caspase-3 H-scores in the lenses and the statistical differences between the groups are shown in Figure 4 and Figure 5. While the mean PCNA H-scores were significantly lower in HC compared to C (*p* < 0.05), the mean caspase-3 H-scores were higher (*p* < 0.05). The differences between the YM-treated groups and HC varied depending on the dose.

### 3.5. Genetic Analysis

**Figure 6:**
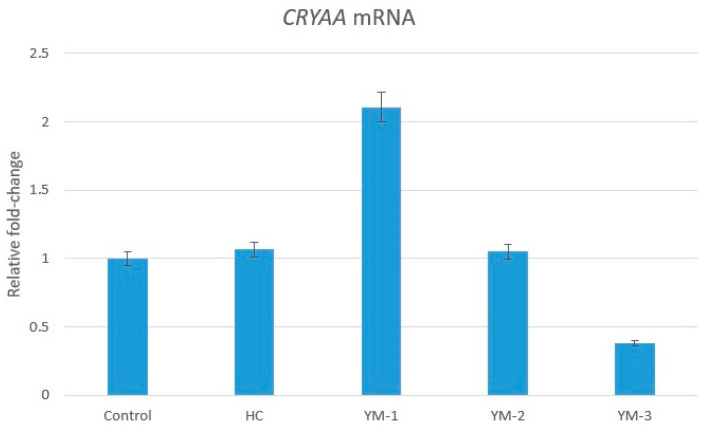
*CRYAA* gene mRNA relative fold-change regulation in embryo lenses treated with HC and different doses of YM. The *GAPDH* gene was used for normalization. **HC:** hydrocortisone group, **YM:** yerba mate aqueous extract, **YM-1:** YM 62.5 mg/kg group, **YM-2:** YM 125 mg/kg group, **YM-3:** YM 250 mg/kg group.

*CRYAA* expression in the lenses of HC and YM-2 embryos was approximately equal to the control level (1.07- and 1.05-fold, respectively). *CRYAA* expression was upregulated in the lenses of YM-1 embryos compared to C (2.11-fold). In contrast, *CRYAA* expression was downregulated (0.38-fold) in the embryonic lenses of YM-3 compared to C (*p* > 0.05) (Figure 6).

## 4. Discussion

There is a growing interest in the discovery of bioactive compounds with therapeutic and nutraceutical properties. YM, a remarkable plant in this context, is a valuable plant that can be used for the development of new foods, nutraceuticals, or drugs [26,27,28,29]. Recent studies have shown that consumption of YM has antioxidant [30], anti-inflammatory, immunomodulatory [31], and chemopreventive [32] effects and can also reduce cardiovascular and diabetes risks [28]. Previous studies have investigated the therapeutic and protective effects of phytochemicals and plant extracts with antioxidant activity against oxidative stress and found positive results [2,5,18,20,33,34,35]. In this study, we investigated the potential protective effect of YM aqueous extract at three different doses (62.5, 125, and 250 mg/kg) in a steroid-induced cataract model in chicken embryos. The phytochemical analyses showed that secondary metabolites such as chlorogenic acid, hesperidin, rutin, and quinic acid were present in high concentrations. Previous studies have reported that YM is rich in bioactive components with high antioxidant activity, which is also consistent with our study [36,37,38,39,40]. Moreover, an increase in antioxidant capacity and markers (GSH, catalase, superoxide dismutase) and a decrease in lipid peroxidation markers (MDA) of YM (3 × 750 mg, orally for 60 days) were found in a study on healthy volunteers (n = 14) [41]. In our cataract model, treatment with YM was found to reduce mean cataract grade at all doses. Our findings are consistent with the results of previous studies, although the experimental models in which cataracts were generated, and the antioxidant treatments applied, were different [2,17,18,20]. In our study, in addition to cataract morphology, the effects of YM treatment on oxidative stress (TAS, TOS, GSH, MDA), apoptosis (caspase-3), and proliferation (PCNA) were investigated.

Oxidative stress and lipid peroxidation are important factors triggering cataractogenesis [1,33,42]. Lipid peroxides can impair the permeability of cell membranes, causing pathological changes in the internal composition and configuration of cells. Together with the loss of protein function, this ultimately leads to the formation of cataracts [43,44]. This has prompted many researchers to investigate the cataract-preventive effect of exogenous antioxidants on cataracts using various models. The beneficial effects of various antioxidants such as ginsenoside-Rb1 [20], lycopene [45], piperine [2], betaine [19], astaxanthin [17], and vitamin E [18] against selenite/HC-induced cataracts in chicken embryos have been reported.

In evaluating the protective effect of YM in our study, we measured the activities of TAS, TOS, GSH, and MDA, which are the basic biochemical markers for detecting oxidative stress status and lipid peroxidation. In this study, the application of HC decreased the levels of TAS and GSH in the lenses, while the levels of TOS and MDA increased. This result can probably be explained by the HC-induced increased oxidative stress and decreased antioxidant capacity, as in previous studies [2,20]. Similar to the results of other antioxidant agents, YM-2 was found to neutralize the effect of HC on GSH and MDA and protect the lens from oxidative damage by reversing the effect of HC on oxidative stress parameters [20,45]. In in vitro, in vivo, and clinical studies, YM has been reported to have potent antioxidant effects that reduce the likelihood of oxidative stress-induced diseases, which is consistent with this study [26,37,38,46,47]. On the other hand, unlike YM-2, YM-3 was unable to regulate the decrease in GSH and the increase in MDA levels by HC (*p* > 0.05). It was hypothesized that this situation could be related to the potential toxicity of high doses of the extracts. In addition to the health-promoting effects of YM, there are also reports of its toxicity. In studies conducted in countries where YM consumption is well above mean, consumption of this beverage has been statistically associated with an increase in the incidence of various cancers such as esophageal, pharyngeal, and laryngeal cancer [26]. Studies show that exposure to toxic polycyclic aromatic hydrocarbons (PAHs), which can be caused by the consumption of YM, increases the risk of disease [48,49,50]. PAHs can accumulate in YM due to environmental pollution, but also because the plant is exposed to high temperatures during drying or extraction preparation [26,50]. Differences in benzo[α]pyrene (human carcinogen) levels in YM infusions may be due to differences in the PAH content of the raw material and the methods used to prepare the infusions, such as the ratio of dry leaves to water, the water temperature, or the infusion time [51]. In this study, the YM extract was prepared by the infusion method, which is the most commonly used method. Considering our results and the findings in the literature that YM may have carcinogenic potential due to increased PAH content when prepared hot, modification of the YM preparation procedure could reduce the potential risk and make the powerful nutraceutical/therapeutic use of YM safe [52].

There are only a limited number of studies in the literature investigating the effect of YM extract on eye diseases. Tate et al. [53] have shown in vitro that YM extract prevents the consequences of oxidative stress such as cell death and premature aging in retinal pigment epithelial cells. In their most recent study, the same study team reports that YM aqueous extract (400 mg/mL) prevents in vivo damage to the retinal pigment epithelium and photoreceptors in a model of age-related macular degeneration associated with oxidative stress in mice [54]. The protective effect of YM extract on organs such as the heart and brain as well as the eye has already been reported [21,55]. Schinella et al. [21] investigated the antioxidant and cardioprotective effects of YM aqueous extract in isolated rat hearts. The result of the study was that the application of YM protected the myocardial tissue of rats from oxygen free radical damage due to ischemia and reperfusion. It was also reported that the amount of MDA was reduced and GSH levels were maintained by the YM extract. Colpo et al. [55] investigated the protective effect of YM extract on the oxidative damage in the brain caused by chronic immobilization stress (6 h of inactivity for 21 consecutive days in rats). While an increase in oxidized lipid and carbonyl levels was observed in all brain regions during chronic immobilization stress, lipid and protein oxidation was found to decrease in the group receiving YM extract compared to the control group. In addition, it was found that GSH/GSSG balance tended to increase in all regions. The study shows that YM extract taken orally (with food) protects against stress in neurons. Furthermore, in a study evaluating the antioxidant effect of YM (20 mg/kg/day, by gavage) on 16-month-old female Wistar rats during perimenopause, it was found that when oxidative stress increased, total plasma antioxidant capacity (FRAP) increased. While a significant decrease in MDA levels was observed in both erythrocytes and liver tissue when YM was administered, without estrogen levels being affected by YM, a significant decrease in superoxide dismutase and glutathione peroxidase levels was observed [56].

The epithelial cells of the lens ensure the stability and transparency of the environment inside the lens. ROS have been reported to induce apoptosis in lens epithelial cells by producing large amounts of additional free radicals that can lead to the degeneration and loss of function of the lens [1]. Furthermore, the stages of cataract formation were investigated in different models, and it was found that proliferation, migration, transdifferentiation, and apoptosis processes were effective in the lens epithelial cells [25]. Therefore, in our study, we hypothesized that the disturbed balance between apoptosis and proliferation might be responsible for the development and progression of cataracts. The immunohistochemical analysis of our study revealed that HC caused a decrease in PCNA, an indicator of proliferation, and an increase in caspase-3, an indicator of apoptosis (*p* < 0.05). On the other hand, it was observed that treatment with YM at relatively low doses (62.5 and 125 mg/kg) regulated caspase-3 and PCNA levels by regulating apoptosis in the lenses and providing significant protection against cataract formation. However, upregulation of YM-3 was unsuccessful in YM-1 and YM-2. We think that this may be related to the possible toxicity of YM described above. When the data obtained are analyzed together, these results suggest that YM may suppress apoptosis by exerting a protective effect against oxidative damage at low and medium doses. We can conclude that this hypothesis can be explained by the fact that YM increases PCNA expression and decreases caspase-3 expression.

The *CRYAA* gene is one of the genes encoding an important structural protein in the lens that plays a role in the development of age-related curved cataracts [57]. The *CRYAA* gene is considered to be a molecular chaperone. It is mainly found in the lens, and mutations in the *CRYAA* gene lead to recessive or dominant cataracts [58]. This gene has been shown to be reduced in patients with age-related cataracts compared to healthy controls [59]. While in the present study, *CRYAA* expressions were similar in HC and YM-2, an increase in *CRYAA* expression of about twofold was observed in YM-1. A significant decrease in *CRYAA* gene was observed in YM-3 compared to C. The expression of this *CRYAA* gene is lower in chickens than in other animals [60]. In addition, many different variants and mechanisms as well as the *CRYAA* gene may play a role in congenital or age-related cataracts [61]. The increased expression of *CRYAA* in YM-1 in our study suggests a possible effect through the reduction of oxidative stress and apoptosis in cataracts. The results of our genetic analysis support the findings that YM-1 has positive effects on cataract pathogenesis, YM-2 is ineffective, and YM-3 may have negative effects due to potentially toxic metabolites.

It has been suggested that YM may also have a positive side effect on diabetes [62]. Positive effects of YM extract were observed in mice fed a high-fat diet and in diabetic mice [63,64]. YM could also be beneficial in metabolic complications of diabetes such as cataracts.

We are aware that our study has some limitations. The present results refer to cataracts and may not be representative for all experimental cataract models. Depending on the type of cataract, different results may occur. On the other hand, toxic PAHs, which may form when hot water is used in the extract prepared by the traditional method, may have prevented the therapeutic efficacy of high doses. Our results emphasize the need to educate consumers about the preparation method.

## 5. Conclusions

In summary, this is the first study to show that relatively low doses of YM (62.5 and 125 mg/kg) reduce cataract development by reducing oxidative stress and apoptosis. On the other hand, high doses of YM (250 mg/kg) were found to be unable to neutralize the effects of HC. The observation of non-dose-dependent effects suggests that YM may be effective in preventing cataract formation and is beneficial even at low doses. This is important in terms of reducing potential side effects and safe use. It was predicted that YM could play a promising role in protecting the lens in age-related and oxidative stress-induced cataracts. The current results pave the way for epidemiologic and clinical research on YM tea, a nutraceutical. However, comprehensive toxicity testing is recommended to determine the safe dose profile of YM’s potential protective effect on cataracts or various organs.

## Figures and Tables

**Table 1:** Primer sequences.

Gene	Primer Sequences (5′ → 3′)
*CRYAA-Forward*	GACCACGGCTACATCTCTCG
*CRYAA-Reverse*	TGGTGTTACTCGTGCACTCC
*GAPDH-Forward*	CTCAACGGATTTGGCCGTAT
*GAPDH-Reverse*	AATGCCAAAGTTGTCATGGATG

The primer sequences of *CRYAA* (>NM_001030797.2 Gallus gallus crystallin alpha A) and *GAPDH* (>NM_204305.2 Gallus gallus glyceraldehyde-3-phosphate dehydrogenase) were designed based on the FASTA format from NCBI. [https://www.ncbi.nlm.nih.gov/nuccore/NM_001030797.2/] (accessed on 23 July 2024).

**Table 2:** LC-MS/MS data of compounds detected in yerba mate aqueous extract.

No.	Analyte	YM (mg/g)	No.	Analyte	YM (mg/g)	No.	Analyte	YM (mg/g)	No.	Analyte	YM (mg/g)
1	Quinic acid	6.847	15	Epicatechin	n.d.	29	Salicylic acid	0.051	43	Nicotiflorin	2.368
2	Fumaric aid	n.d.	16	Vanilic acid	n.d.	30	Cyranoside	n.d.	44	Fisetin	n.d.
3	Aconitic acid	n.d.	17	Caffeic acid	0.403	31	Miquelianin	n.d.	45	Daidzein	n.d.
4	Gallic acid	n.d.	18	Syringic acid	n.d.	32	Rutin-D3-IS ^a^	n.a.	46	Quercetin-D3-IS ^a^	n.a.
5	Epigallocatechin	n.d.	19	Vanillin	0.035	33	Rutin	7.619	47	Quercetin	n.d.
6	Protocatechuic acid	0.084	20	Syringic aldehyde	n.d.	34	Isoquercitrin	0.450	48	Naringenin	n.d.
7	Catechin	n.d.	21	Daidzin	n.d.	35	Hesperidin	8.166	49	Hesperetin	n.d.
8	Gentisic acid	n.d.	22	Epicatechin gallate	n.d.	36	O-Coumaric acid	n.d.	50	Luteolin	n.d.
9	Chlorogenic acid	23.723	23	Piceid	n.d.	37	Genistin	n.d.	51	Genistein	n.d.
10	Protocatechuic aldehyde	0.210	24	*p*-Coumaric acid	n.d.	38	Rosmarinic acid	n.d.	52	Kaempferol	n.d.
11	Tannic acid	n.d.	25	Ferulic acid-D3-IS ^a^	n.a.	39	Ellagic acid	n.d.	53	Apigenin	n.d.
12	Epigallocatechin gallate	n.d.	26	Ferulic acid	n.d.	40	Cosmosiin	n.d.	54	Amentoflavone	n.d.
13	1,5-dicaffeoylquinic acid	n.d.	27	Sinapic acid	n.d.	41	Quercitrin	n.d.	55	Chrysin	n.d.
14	4-OH benzoic acid	n.d.	28	Coumarin	n.d.	42	Astragalin	0.094	56	Acacetin	n.d.

^a^ IS: internal standard, YM: yerba mate aqueous extract, n.d.: not detected, n.a.: not applicable.

**Table 3:** Grades of cataract of the lens samples in all study groups.

Groups	Cataract Grades (n)					n	*p*-Value
	1	2	3	4	5		
**C**	16	0	0	0	0	16	<0.05
**HC**	0	0	3	11	2	16	
**YM-1**	0	0	3	11	2	16	
**YM-2**	0	13	3	0	0	16	
**YM-3**	0	3	10	3	0	16	
**Total**	16	23	20	19	2	80	

**C:** control group, **HC:** hydrocortisone group, **YM:** yerba mate aqueous extract, **YM-1:** YM 62.5 mg/kg group, **YM-2:** YM 125 mg/kg group, **YM-3:** YM 250 mg/kg group.

## Data Availability

Data will be made available on request.

## Supplementary Materials

The following supporting information can be downloaded at: https://www.mdpi.com/article/10.3390/life14080994/s1, Figure S1: The dissociation curve of the real-time PCR.
