# Supplementary figures and images for: Anti-Cataract Effect of the Traditional Aqueous Extract of Yerba Mate (Ilex paraguariensis A. St.-Hil.): An In Ovo Perspective
Source: Life (Basel). 2024 Aug 10;14(8):994. doi: 10.3390/life14080994 (PMC11355064; doi:10.3390/life14080994)

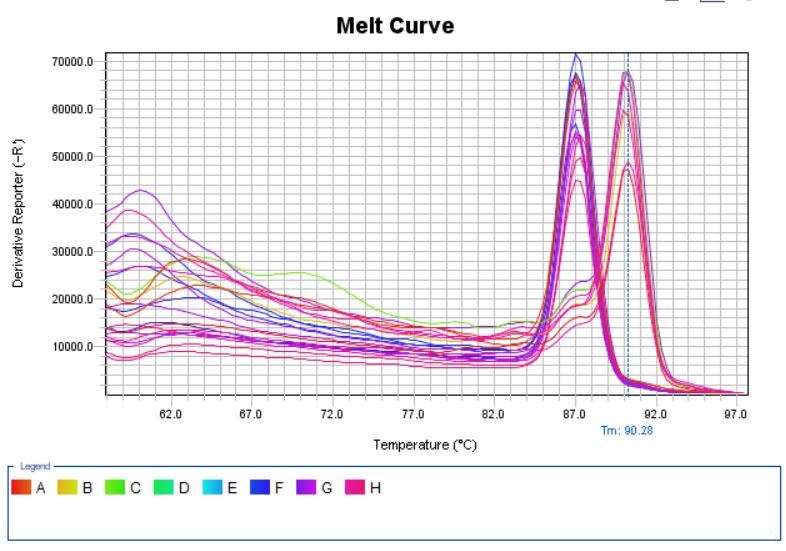

Supplement: Supplementary file 1 [file life-14-00994-s001.zip › life-3095797-Figure S1.JPG]
